# Balanced Crystalloid Use Is Associated With Haemodynamic Stability And Less Need For Vasopressors in Patients Receiving Renal Transplantation Compared To 0.9% Saline

**DOI:** 10.1186/2197-425X-3-S1-A18

**Published:** 2015-10-01

**Authors:** CA Pfortmueller, GC Funk, E Potura, C Reitere, B Kabon, W Druml, E Fleischmann, G Lindner, the Vienna Interdisciplinary Research Group on Fluids, Acid-base Disorders and Electrolytes

**Affiliations:** Vienna General Hospital and University of Vienna, Clinic for General Anesthesiology, Intensive Care and Pain Managment, Vienna, Austria; Otto Wagner Hospital Vienna and Ludwig-Boltzmann Institute for COPD and Respiratory Epidemiology, Department of Respiratory and Critical Care Medicine, Vienna, Austria; Medical University of Vienna, Department of Nephrology, Vienna, Austria; Hirslanden - Klinik im Park, Department of Emergency Medicine, Zurich, Switzerland

## Background

Data is suggesting that non-balanced conventional crystalloids (0.9% saline) are not only associated with the occurrence of hyperchloremic, metabolic acidosis but increased incidence of acute kidney injury and even morbidity and mortality. Experimental data suggested infusion of hyperchloremic crystalloids to be associated with hypotension in experimental sepsis. We aimed to investigate whether use of modern balanced crystalloids lead to better hemodynamic stability compared to 0.9% saline in the perioperative period.

## Methods

We performed a subanalysis of a prospective, randomized, controlled trial comparing effects of 0.9% saline or an acetate-buffered, balanced crystalloid during the perioperative period in patients with end-stage renal disease undergoing cadaveric renal transplantation. Blood pressure and need for catecholamine therapy were the primary measures.

## Results

A total of 150 patients were included in the study. 76 were randomized to 0.9% saline while 74 received an acetate-buffered balanced crystalloid. There was no difference in the volume of fluid administered (2,625 [2,000-3,100] in the saline vs. 2,500 ml [2,000-3,050] in the balanced group; p = 0.83). Chloride was significantly higher in the saline group (109 [107-111] versus 107 mmol/L [105-109]). Mean blood pressure was significantly higher in the balanced group. Mean minimum blood pressure was significantly lower in the saline group (57.2 [SD 8.7] versus 60.3 mmHg [SD 10.2]; p = 0.024). More patients received noradrenaline in the group receiving 0.9% saline (30% versus 15%; p = 0.0278).

## Conclusions

Our data suggest that use of 0.9% saline results in hyperchloremic metabolic acidosis and is associated with a higher incidence of hypotension and vasopressor use in the perioperative period.

